# Amorphous aggregates with a very wide size distribution play a central role in crystal nucleation†

**DOI:** 10.1039/d4sc00452c

**Published:** 2024-07-04

**Authors:** Zhiyu Liao, Ankita Das, Christina Glen Robb, Rebecca Beveridge, Klaas Wynne

**Affiliations:** a School of Chemistry, University of Glasgow G12 8QQ UK klaas.wynne@glasgow.ac.uk; b Dept. of Pure and Applied Chemistry, University of Strathclyde Glasgow G1 1XL UK

## Abstract

There is mounting evidence that crystal nucleation from supersaturated solution involves the formation and reorganization of prenucleation clusters, contradicting classical nucleation theory. One of the key unresolved issues pertains to the origin, composition, and structure of these clusters. Here, a range of amino acids and peptides is investigated using light scattering, mass spectrometry, and *in situ* terahertz Raman spectroscopy, showing that the presence of amorphous aggregates is a general phenomenon in supersaturated solutions. Significantly, these aggregates are found on a vast range of length scales from dimers to 30-mers to the nanometre and even micrometre scale, implying a continuous distribution throughout this range. Larger amorphous aggregates are sites of spontaneous crystal nucleation and act as intermediates for laser-induced crystal nucleation. These results are shown to be consistent with a nonclassical nucleation model in which barrierless (homogeneous) nucleation of amorphous aggregates is followed by the nucleation of crystals from solute-enriched aggregates. This provides a novel perspective on crystal nucleation and the role of nonclassical pathways.

## Abstract 2

There is mounting evidence that crystal nucleation from supersaturated solution involves the formation and reorganization of prenucleation clusters, contradicting classical nucleation theory.

## Graphical abstract

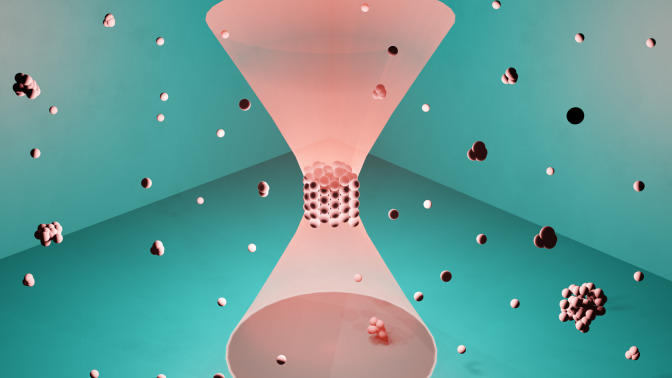

## Introduction

The nucleation of crystals from solution is traditionally described in the framework of classical nucleation theory, in which the key criterion is the critical size of a nucleus growing by the attachment of individual solute molecules. However, classical nucleation theory has been challenged by observations of nanoscale and mesoscale metastable solute species in super- and even undersaturated solutions without initiating crystal growth.^1–3^ Accordingly, alternative mechanisms, collectively referred to as nonclassical nucleation theory, were proposed in which nucleation involves the formation and assembly of prenucleation clusters, liquid droplets, or other amorphous oligomeric complexes.^1,2,4–6^ Such pathways were discovered in systems that are close to a liquid–liquid phase transition,^7–9^ and subsequently, many more examples have been found of apparently amorphous intermediates or mesoscale aggregates in solution playing a role in crystal nucleation of organic^3,10–12^ and inorganic molecules,^13–20^ proteins,^21^ and laser-induced crystal nucleation.^22–25^ The radius of such clusters may range from 75 nm to as much as 500 nm.^24,26^ The relative stability of these aggregates (and lack of macroscopic phase separation) would seem to be inconsistent with liquid–liquid phase separation. The investigation of the nature and behaviour of amorphous aggregates is of great importance to the fundamental understanding of crystal nucleation.

The crystallization and amorphous phases of amino acids and small peptides are of particular interest due their application in peptide-based supramolecular materials,^27^ engineered amino acid crystals with special physical properties,^28^ and peptide-based drugs,^29^ where crystallization is key to unlocking pharmaceutical applications.^30^

Here, we show that a range of amino acids and a number of di- and tripeptides in supersaturated aqueous solution also form amorphous aggregates and investigate their role in laser-induced and spontaneous crystal nucleation. They form on a timescale of approximately one day and redissolve on a timescale of hours, while *in situ* Raman spectroscopy confirms their amorphous nature. Using dynamic light scattering, we demonstrate that these aggregates are far from monodisperse but have a wide range of sizes consistent with a very wide size distribution. Mass spectrometry is used to confirm that the solute molecules cluster over a very wide range of sizes from dimers to 30-mers and greater, suggesting a continuous distribution from the molecular to the micrometre scale. Most of the samples investigated show aggregate-assisted laser-induced nucleation. In several cases, the amorphous aggregates could be established as sites of slow spontaneous crystal nucleation. These results indicate a universal role of amorphous aggregates in crystal nucleation. We will show that classical nucleation theory can be amended using the concept of fractal aggregates with a reduced interfacial tension and a free energy of formation that decreases with increasing size. This simple modification explains the observed wide size distribution and the presence of amorphous aggregates even in undersaturated solutions. The much-increased supersaturation inside the aggregates allows for spontaneous as well as laser- or shearing-induced crystal nucleation. This provides a new unified understanding of the nucleation of molecular aggregates and crystals. It will have wider application to other aggregation phenomena, such as that of G-quadruplexes^31^ and aggregation–induced emission,^32^ and implications for the development of amorphous drugs.

## Results and discussion

### Formation and dissolution of aggregates

Numerous studies have described the formation of chain-like structures, mesoscale clusters, amorphous nanoparticles, transient liquid droplets, *etc*. in supersaturated solutions. However, due to the slow rate of formation and nonequilibrium nature, it is unclear whether these aggregates have the expected thermodynamic behaviour of nucleation and redissolution. Therefore, we carried out dynamic light scattering studies (DLS, see Materials and methods for details) on a range of amino acids and peptides to elucidate the formation and redissolution of aggregates in solution on ultraslow timescales.

**Fig. 1:**
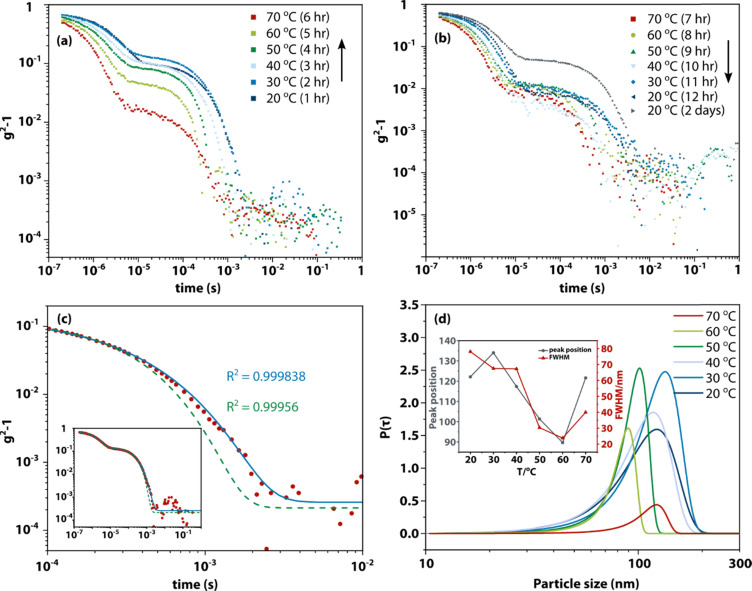
Dynamic light scattering showing the presence of nanometre aggregates in supersaturated solution, dissolution upon heating, and reappearance upon cooling. Experimental dynamic light scattering intensity autocorrelation functions with the baseline subtracted of Gly–Gly–Gly (0.1 g mL^−1^) as a function of temperature. (a) Solution aged for 1 day slowly warmed up (1 hour equilibration at each temperature) to induce dissolution. (b) Same sample cooled after the heating experiment shown in (a). (c) Comparison of standard exponential fitting and stretched exponential fitting to the intensity autocorrelation function at 20 °C shown in (a). Red dots: experimental intensity autocorrelation function; green dashed line: fit with an exponential function; blue solid line: fit with a stretched exponential function. (d) Particle size distribution of the slower components in the decays shown in (a), based on stretched exponential fitting (see Table S1† for fit parameters), *P*(*τ*) denotes the integrated probability. Inset shows the changes of peak position and width (full width at half maximum) as function of temperature.

Fig. 1 shows an example of the DLS measurement of the formation and dissolution of aggregates of glycine tripeptide (Gly–Gly–Gly) in aqueous solution as a function of temperature. The solution was prepared at 80 °C at a concentration of 0.1 g mL^−1^ (see Materials and methods for sample preparation and Table S2† for concentrations and solubilities). The intensity autocorrelation curves show that there are two components in the decays: a fast decay (∼10^−6^ s) due to concentration fluctuations of solute molecules and a much slower decay (∼10^−3^ s) due to the aggregates. Upon slowly raising the temperature from 20 °C to 70 °C, the slow decay gradually weakens. This unambiguously demonstrates the redissolution of the aggregates at higher temperatures. On subsequent cooling back to 20 °C at the same rate, the slow decay is gradually restored but not to the starting level. Only after ageing the solution for another two days is the slow decay amplitude fully restored. This demonstrates that the aggregates that can be observed by DLS form extremely slowly in supersaturated solution (consistent with literature reports) but otherwise redissolve normally. In general, fresh solutions do not tend to nucleate crystals, while aged solutions, having formed aggregates, eventually nucleate crystals after a day or days. This shows that the aggregates are metastable with respect to crystallization and facilitate crystal nucleation.

The hydrodynamic diameters and size distributions obtained by analysing the intensity autocorrelation functions using cumulant analysis (using software that comes with the instrument) are shown in Fig. S1.† The first peak at 0.97 nm is consistent with the size of Gly–Gly–Gly. The second peak near 100–200 nm is more variable in position and has a height that decreases with increasing temperature, as expected. These results are broadly consistent with previous studies on similar solutions.^24,33^

However, it is highly surprising that aggregates—which are thermodynamically more stable than the supersaturated solution—would grow to a size of 100–200 nm and then stop, especially since the component parts—molecules—are only ∼1 nm. This suggests that the solutions should contain a range of aggregate sizes that is missed by a simple DLS experiment.

To test this idea, the DLS intensity correlation functions were instead analysed using a stretched exponential function, e^−(*t*/*τ*)^*β*^^. Much better fits could be obtained using this function (see Fig. 1(c)). Fig. 1(d) shows the particle size distributions (see Materials and methods) obtained by fitting the temperature-dependent data with stretched exponential functions. These distributions are broad (*circa* 80–200 nm) at low temperature while sharpening up and moving to lower particle sizes (*circa* 80–100 nm) at higher temperature. Because the dependence of the light-scattering intensity on the 6th power of the particle size has not been included (see below), these distributions imply a very broad particle size distribution.

**Fig. 2:**
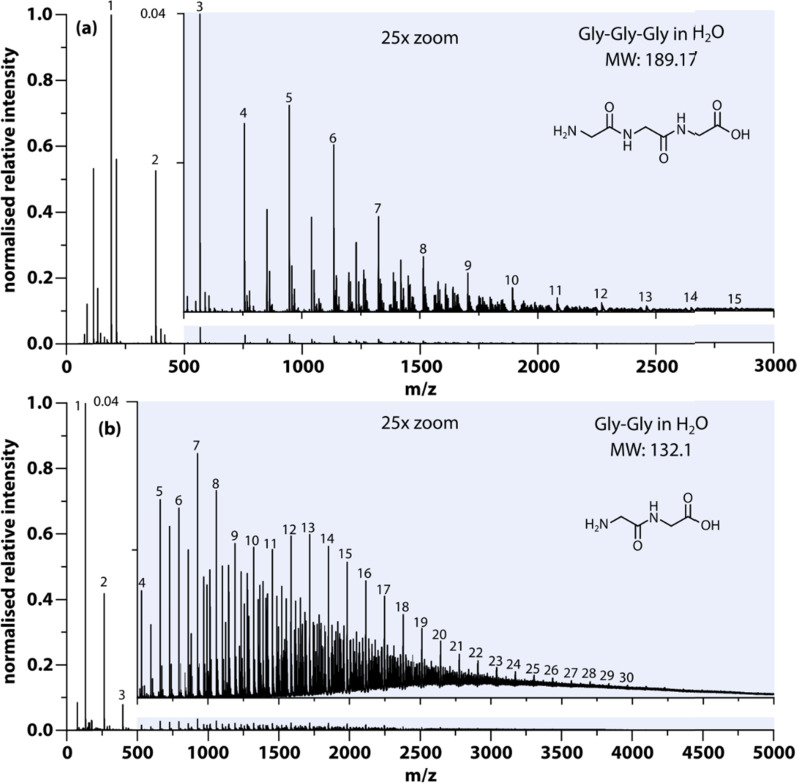
Characterization of small Gly–Gly–Gly and Gly–Gly aggregates using mass spectrometry. Samples were prepared in H_2_O and characterized 4 hours after preparation at 70 °C using mass spectrometry. (a) Detailed spectrum showing mass to charge (*m*/*z*) from 0–3000 of Gly–Gly–Gly (0.1 g mL^−1^); the insert corresponds to the shaded area from 500–3000. Numbers above peaks indicate the size (number of monomers in the oligomer) of oligomers within aggregates for the singly charged species. (b) Similar spectrum showing *m*/*z* from 0–5000 of Gly–Gly (0.2 g mL^−1^).

The results from nanoelectrospray ionization-mass spectrometry also support the idea that aggregates assume a large range of sizes ranging from dimers to oligomers. As an example, Fig. 2(a) shows the size distribution of aggregates formed in Gly–Gly solution (0.2 g mL^−1^). The detailed mass spectra reveal that dimers, trimers, and up to the 30th oligomers can be detected within the signal-to-noise ratio, while the intensity decreases with aggregate size. Peaks with *m*/*z* in between oligomers are due to multiple charging, which likely implies much larger aggregates, but in this study, only the singly charged species have been annotated. Similar results have been obtained in several of the solutions studied here, *e.g.*, Gly–Gly–Gly, as shown in Fig. 2(b).

### The amorphous character of the aggregates

**Fig. 3:**
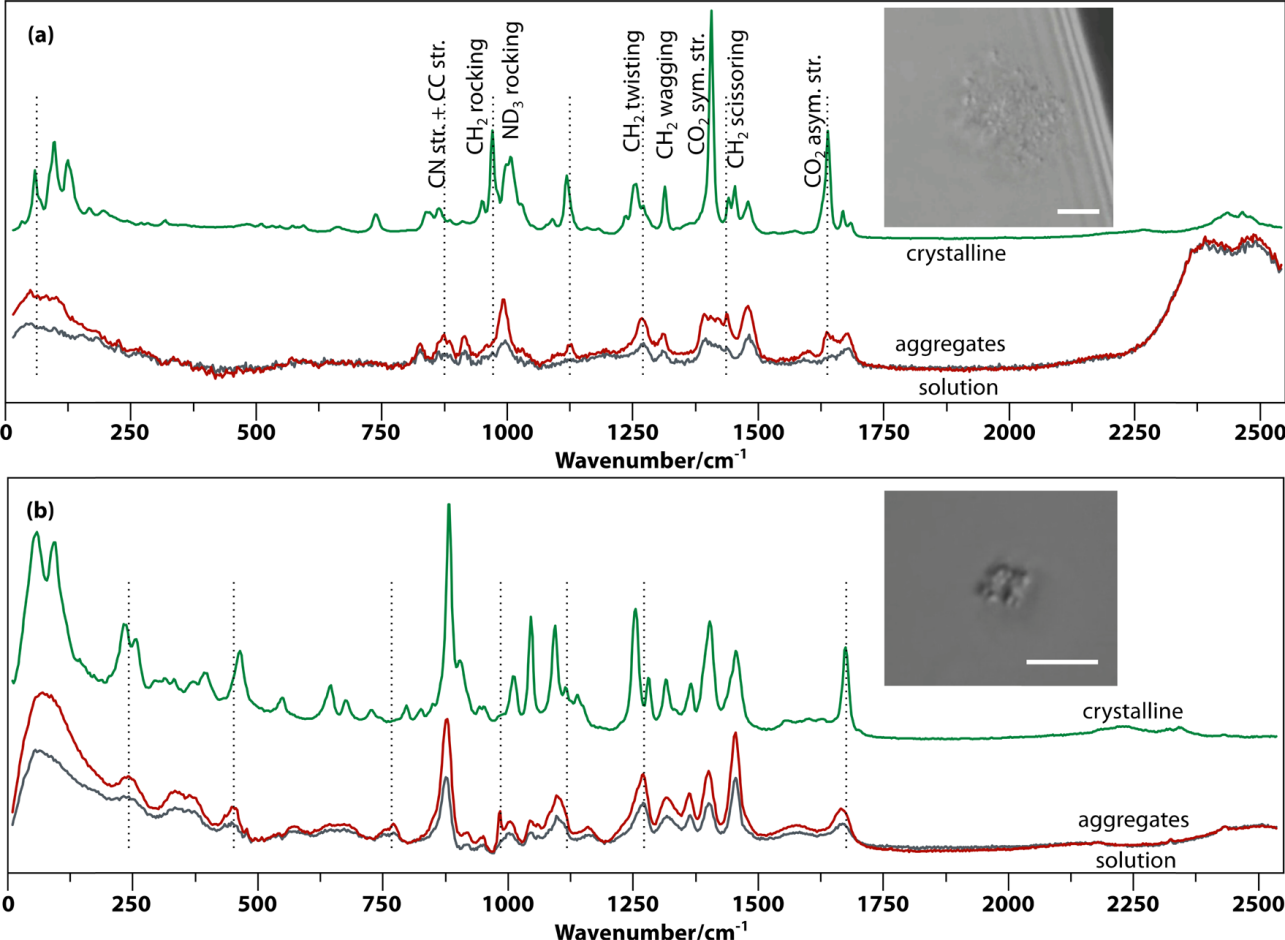
*In situ* Raman spectroscopic characterization of Gly–Gly–Gly and Ala–Ala aggregates in solution demonstrating their amorphous nature. The Raman spectra of the solution in D_2_O, aggregates, and a crystal of (a) Gly–Gly–Gly and (b) Ala–Ala are shown. (Insets) Aggregates in solution observed by phase-contrast microscopy (scale bars, 5 μm).

Raman spectroscopy was employed to characterize the aggregates in solution using a setup that allows optical tweezing and *in situ* confocal Raman microscopy as described previously.^22^ In most of the investigated amino acid and peptide solutions, the aggregates visible by microscopy are small (<1 μm) and rare. In contrast, supersaturated Gly–Gly–Gly and alanine dipeptide (Ala–Ala) solutions form many aggregates after aging, which cluster—assisted by optical tweezing by the Raman excitation laser—to form larger aggregates (see insets of Fig. 3). The *in situ* Raman spectra of the (supersaturated) solution of Gly–Gly–Gly, the aggregates trapped in the laser beam, and the crystal are shown in Fig. 3(a). The spectra of the aggregates and solution are very similar, but the former has approximately double the intensity. This shows that the aggregates are solute rich and are not foreign particles such as dust. At very low frequencies (0–200 cm^−1^), the aggregate spectrum is more well defined than that of the solution; however, it does not show the three sharp phonon peaks of the crystal. This indicates a degree of order in between solution and crystal.

A number of bands in the fingerprint region that are prominent in the crystal are weak in the aggregates. For example, the CH_2_ rocking band (970 cm^−1^) and the CO_2_ symmetric and asymmetric stretching modes (1407 cm^−1^ and 1638 cm^−1^). The CO_2_ asymmetric stretching peak (1638 cm^−1^) is absent or very weak in solution but much more intense in the aggregate while dominating in the spectrum of the crystal. Additionally, less prominent changes are the splitting of the ND_3_ rocking band (993.3 to 993.3/1006.2 cm^−1^), CH_2_ twisting (1269 to 1256.6/1271.6 cm^−1^) and amide I vibration (at 1679 to 1669.6/1683.8 cm^−1^) from aggregate to crystal.

Similar spectral changes have been found in other amino acids and peptides. For example, Fig. 3(b) shows spectra of aggregates formed in Ala–Ala solution. Here, the aggregates show much stronger Raman intensity and some distinct peaks (*e.g.*, 984.2 cm^−1^) compared to solution, as well as peak coalescence and shifting compared to the crystal (*e.g.*, 242.4, 878, 1096.7, and 1665.5 cm^−1^). The very low frequency spectrum (0–200 cm^−1^) shows a broad featureless band, suggesting even less ordering in Ala–Ala compared to Gly–Gly–Gly aggregates. Another example is that of threonine aggregates (Fig. S2†), which show prominent and distinctive fingerprint peaks compared to solution.

### The role of aggregates in laser-induced crystal nucleation

In previous work, we have shown that aggregates in supersaturated glycine solution are an intermediate to laser-induced crystal nucleation^22^ but could not demonstrate the generality of the effect. Here, we studied nine amino acids and three peptides (see Table S2† for concentrations and solubilities), found aggregates in all but one (arginine, with a pH value of 11.63 after preparation, but aggregates were also not observed at an adjusted pH of 4.5 or 13), and observed laser-induced nucleation in nine of these (see movies S1–S9†).

A few examples will be discussed here, where a 50 mW 532 nm CW laser was employed for laser-induced nucleation and optical trapping and as the Raman excitation source. Fig. S3(a)† shows laser-induced nucleation of alanine from supersaturated aqueous solution that has been aged for 2 days (see also movie S1†), followed by *in situ* Raman spectroscopy. Due to the small size of the aggregates in this case, the Raman spectrum of the aggregate (*t* = 2 s) is weak and difficult to distinguish from that of the solution. Hence, the amorphous nature cannot be confirmed. However, once laser-induced nucleation takes place (*t* = 4 s), a burst of emission is observed (peaking at ∼580 nm) that is also observable in microscopy. This effect is likely due to crystalloluminescence, in which dopant metal cations trapped in the growing crystal lattice relax through light emission.^34^

Another example of laser-induced nucleation of a supersaturated Gly–Gly solution is shown in Fig. S3(b),† as well as movie S2.† Despite the small size of the aggregate formed (<1 μm, indicated by the arrow in the micrograph), the intensity of the Raman spectrum over the entire range is visibly stronger than that of the surrounding solution (∼× 1.6, see the comparison of spectra at *t* = 3 s and solution) without any detectable difference in peak positions or heights. When brought into the laser focus, nucleation from the aggregate starts almost immediately, with a sizable crystal forming within seconds. The transition from aggregate to crystal is accompanied by peak shifts of C–C-stretch (879 cm^−1^), amide III (1274.4 cm^−1^), and amide I (1687.4 cm^−1^). Unlike the laser-induced nucleation of glycine we reported previously,^22^ no intermediate states could be detected during the transition here despite the good signal strength.

Finally, Fig. S3(c)† shows laser-induced nucleation in a supersaturated solution of Gly–Gly–Gly (movie S3†), where again crystalloluminescence is observed (in microscopy only, showing that the emission is outside the Raman spectral range). The gradual appearance and sharpening of phonon peaks in the low-frequency region (0–400 cm^−1^) shows the process of transition from disordered aggregate to partially ordered intermediate and, finally, to the crystal at *t* = 89 s. Before this point, the peaks in the fingerprint region increase in intensity but otherwise do not change in position or relative intensity. At *t* = 89 s, the morphology changes in microscopy and more drastic changes in the spectrum are observed.

### The role of aggregates in spontaneous crystal nucleation

**Fig. 4:**
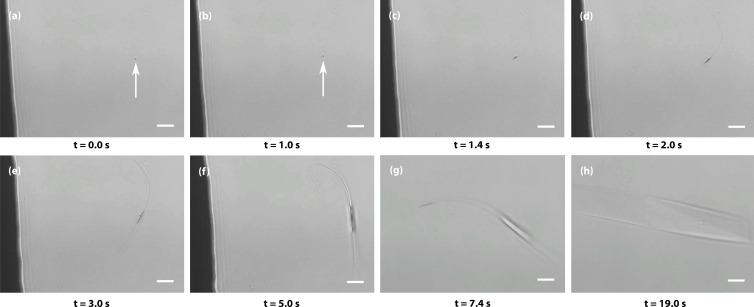
The amorphous aggregates are the site of spontaneous nucleation. Spontaneous crystal nucleation through slow evaporation in aged and supersaturated Gly–Gly–Gly solution. (a–h) Selected microscopic frames at times before and after nucleation started at or within the aggregate indicated by the arrow. Scale bars, 20 μm.

To investigate the role of amorphous aggregates in spontaneous nucleation, evaporation-driven nucleation experiments were carried out and monitored under a microscope. A 1 μL droplet of a supersaturated solution of Gly–Gly–Gly aged for more than 2 weeks was placed on a Petri dish and covered. Micrometre-sized aggregates could be observed and—as the sample slowly evaporated over a few minutes—some of the aggregates initiated spontaneous crystal nucleation. An example is shown in Fig. 4, where changes in the aggregate can be observed from *t* = 1 s when whip-like crystalline structures form at opposite ends of the aggregate, which itself grows and then transforms into a well-shaped crystal (see movie S10† for the entire process). In most repeat experiments, only a small fraction of a large number of aggregates initiates spontaneous crystal nucleation, demonstrating the stochastic nature of the process. (However, in one example, the majority of the aggregates transformed into crystals simultaneously, see movie S11†). Due to the limited spatial resolution of visible-light microscopy, we cannot distinguish between homogeneous crystal nucleation inside the aggregate and heterogeneous nucleation on the surface of the aggregate.

### Revision of classical nucleation theory for fractal aggregates

Although classical nucleation theory is often criticized for not being quantitative, its basic principles would appear to be valid, namely, that the interface is energetically less favourable than the bulk, resulting in a barrier to nucleation. Here, we will show that classical nucleation theory can be amended by taking into account the nature of amorphous aggregates.

**Fig. 5:**
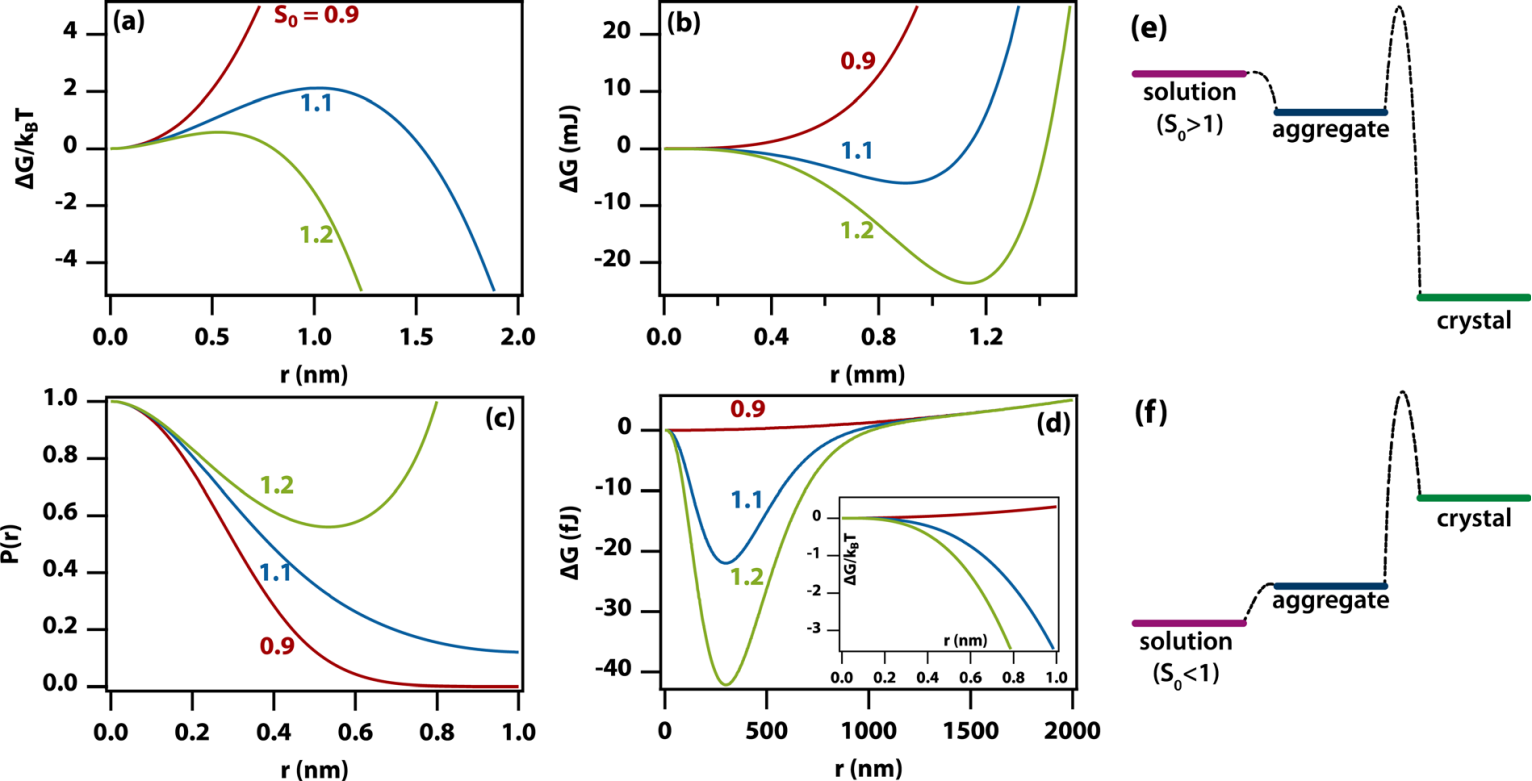
A modified classical theory for the nucleation of amorphous aggregates explains the origin of the very wide aggregate size distribution. (a) The textbook Gibbs free energy curves (including the reduction in supersaturation due to a growing crystal and divided by *k*_B_*T*) as a function of crystal radius in the region near the peak of the barrier for the nucleation of a crystal from supersaturated solution. (Parameters *S*_0_ = 0.9, 1.1, and 1.2 (red, blue, green), *T* = 298 K, *Ω* = 0.1 nm^3^, *γ* = 2 mJ m^−2^, *C*_s_ = 1 M, *V* = 1 mL). (b) Same as (a) but in the region near the free-energy minimum. (c) Probability distribution functions calculated from the Gibbs free energy curves in (a). (d) Gibbs free energy curves for an amorphous aggregate by taking into account a reduction in the free energy of formation with increasing aggregate size. (Parameters as in (a) except *S*_0_ = 1.0, 1.1, and 1.2 (red, blue, green), *γ* = 0.1 mJ m^−2^, *δ* = 0, *λ* = 100 nm). The inset shows that the free energy does not have a barrier for the formation of aggregates. (e) Relative free energies of a supersaturated solution, aggregate, and crystal. Formation of the aggregate is barrierless (homogeneous), but formation of the crystal from the aggregate still has a barrier. (f) As in (e) but for an undersaturated solution. As aggregate formation is barrierless, there should be a (small) number of aggregates even in undersaturated solutions.

There are many excellent reviews of classical nucleation theory,^35^ and in the following, only the basic premise of the theory will be described. The equation for the change in free energy for a spherical nucleus of radius *r* can be written in terms of the supersaturation as1
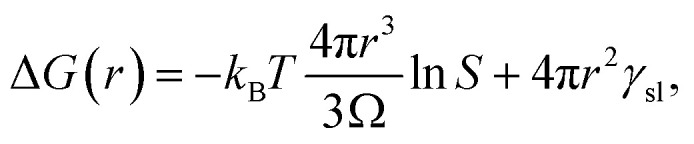
where *Ω* is the molecular volume in the crystal, *S* is the supersaturation, and *γ*_s*l*_ is the surface tension. The supersaturation is here defined by *S* = *C*/*C*_S_, where *C* is the concentration of the solute and *C*_S_ is the saturation concentration or solubility of the crystal. The molecular volume in the crystal can be calculated from *Ω* = *M*/(1000*ρN*_A_) (in m^3^ per molecule), where *M* is the molar mass (in g mol^−1^) and *ρ* is the density of the crystal (in kg m^−3^). This equation gives the well-known curves shown in Fig. 5(a) with the appearance of a barrier to nucleation, as discussed extensively in the literature. This expression for the free energy has an obvious defect in that it tends to negative infinity as the radius cubed.

However, as the crystal nucleus grows, the supersaturation will decrease from an initial value, *S*_0_. It is straightforward to determine that the radius-dependent supersaturation is given by2
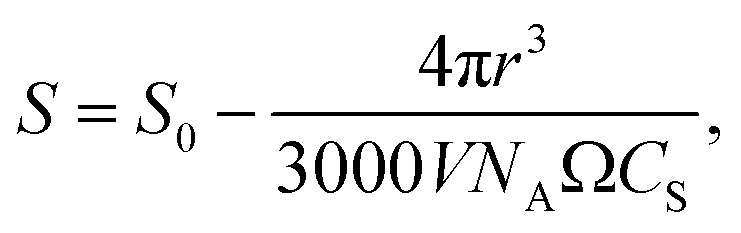
where *V* is the volume of the sample. When including this effect (see Fig. 5(b)), one would intuitively expect the free energy to minimize for a crystal of such a size that the supersaturation has reduced to *S* = 1. This turns out to be not quite the case: the free energy returns to zero at *S* = 1, while the minimum occurs quite a bit before this. Thus, in Fig. 5(b) for *S*_0_ = 1.2, the minimum occurs for a crystal of radius *r* ≈ 1.15 mm where *S* = 1.09, while *S* = 1 only occurs at *r* = 1.42 mm.

Using this expression for the free energy, eqn (1) and (2), the probability distribution can be calculated from *P*(*r*) = exp(−Δ*G*(*r*)/*k*_B_*T*) as shown in Fig. 5(c). The width of this distribution (for parameters valid for a small organic molecule such as glycine) is approximately 0.3 nm, which is on the order of a molecular diameter. In a continuum theory such as this, that simply means that solute molecules are most likely to occur in solution as monomers.

### Fractal aggregates

Amorphous aggregates do not have an ordered arrangement of molecules as the crystal does. Therefore, the enthalpy of formation is reduced, but the entropic penalty is also reduced. Therefore, it is impossible to predict whether the free energy of the aggregate is above or below that of the crystal. Experimentally, in the supersaturated solutions studied here, the amorphous aggregates are metastable with respect to the crystal, implying a higher Gibbs free energy for the former (see Fig. 5(e)). As the supersaturation depends on the change in free energy, this implies that the supersaturation for going from solution to aggregate is not as high as that for going from solution to crystal. As the entropic penalty is also reduced at the interface, the surface tension is expected to be lower, implying a reduction in the barrier to nucleation of aggregates.

In general, there is no direct relation between the supersaturation for going from solution to crystal to that for going from solution to aggregate. However, specific interactions that stabilize the crystal (*e.g.*, hydrogen bonds) are likely to play a role in stabilizing the aggregate as well but entropic contributions are different. In Fig. 5(e) and (f) it has been assumed that the Gibbs free energy of the aggregate is simply in between that of solution and crystal but this is likely an oversimplification.

As the amorphous aggregates are not crystalline, are at most partially structured, and contain significant numbers of solvent molecules, they are expected to have a fractal^20,36,37^ or more generally a disordered structure.^38^ Indeed, this is borne out by experimental observation by microscopy and Raman spectroscopy (see Fig. 3). Therefore, the free energy of formation is expected to decrease as the aggregate becomes larger. Here, we propose that the free energy of formation can be written as3
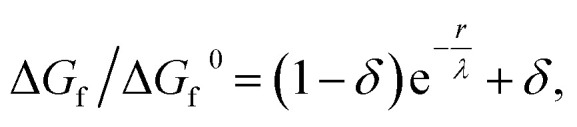
where *λ* is the (fractal) correlation length and *δ* accounts for the possibility that the free energy of formation decays to a finite value. Not enough is known about amorphous aggregates to estimate the fractal correlation length. However, the correlation length in somewhat similar *J* aggregates has previously been estimated to be several hundred molecules,^39^ which would correspond to *λ* ∼100 nm.

Fig. 5(d) shows the change in free energy with nucleus size calculated by using eqn (3) (see ESI†). The parameters used are the same as before except the supersaturation has been reduced from 1.2 to 1.1, the surface tension reduced by a factor of 10, *δ* = 0, and *λ* = 100 nm. For these parameters, the barrier to nucleation (of aggregates) has disappeared. For *r* ≫ *λ*, the surface tension dominates, resulting in a minimum at ∼300 nm. This minimum is ∼10^12^ times shallower than the minimum in the case of crystallization (see Fig. 5(b)) but still ∼10^6^ times deeper than *k*_B_*T*. Thus, in thermodynamic equilibrium, one expects a narrow probability distribution function corresponding to a single aggregate of a very well-defined size.

## Conclusions

### The character of amorphous aggregates and their role in crystal nucleation

Here, we have shown that solute-rich and amorphous to partially ordered metastable aggregates, with an apparent size of *circa* 100 nm, form after 1 to 5 days in supersaturated solutions of a range of amino acids and peptides. The observation of a wide range of oligomers using mass spectrometry suggests that these aggregates form by the conversion of smaller clusters into larger ones. The increased Raman scattering intensity of the aggregates over the solution proves the solute-rich character of aggregates in solutions of threonine, Gly–Gly, Ala–Ala and Gly–Gly–Gly. The broad unstructured bands in the lattice phonon region (0–400 cm^−1^) are typically different from those in the solutions and cannot be reproduced as the sum of the solution and crystal spectra, suggesting a different but still amorphous phase for the aggregates. The aggregates of the remaining amino acids are either too small or not dense enough to show a difference above the noise level. In the case of proline and alanine, the aggregates are not optically trapped but pushed away instead, making it impossible to take a Raman spectrum. Finally, aggregates of phenylalanine, histidine, lysine, and serine undergo instantaneous laser-induced nucleation, leaving no time for integration of a Raman spectrum with distinguishable features.

The amorphous aggregates have two possible roles in laser-induced (and spontaneous) nucleation: provide an effective local supersaturation greater than the starting solution or act as a heterogeneous nucleation site. In the former scenario, the laser either induces greater order through the optical Kerr effect (however, known to be a small effect^40^) or simply provides enough heat for the aggregate to undergo classical nucleation at a much greater effective supersaturation.

The observation of spontaneous crystal nucleation from amorphous aggregates demonstrates that they play a much more general role than just in laser-induced nucleation. There is mounting evidence to support the idea of the existence of amorphous aggregates as being intermediate (on-path) or inhibiting (off-path) for nucleation of a crystal from a supersaturated solution with or without a laser to trigger nucleation.^1,21,41,42^ This is supported by our observations. We have demonstrated that amorphous intermediates are found in aqueous solutions of a range of amino acids and a number of peptides, supporting the idea that it is a general phenomenon. The existence of amorphous aggregates also provides an explanation for the phenomenon of shear-induced nucleation of small-molecule solutes,^43^ as the effect of shear is much greater on a cluster than on a single molecule.

### The very wide size distribution of the amorphous aggregates

Previous studies of amorphous aggregates or droplets using dynamic light scattering (DLS) reported hydrodynamic radii of 75 or 500 nm.^24,26^ Such values are surprising, as the molecular dimensions are approximately 1 nm. If the aggregates were, for example, some sort of micellar structure, it is difficult to understand why growth would stop at 75 nm.

DLS is an excellent technique for determining the size of particles in suspension when the particle size distribution is narrow. Standard DLS analysis software assumes that this is the case. However, here, we find that the experimental DLS intensity-correlation function is modelled much better by fitting to a stretched exponential function, implying a distribution of aggregate sizes, as shown in Fig. 1(d). However, the light scattering efficiency scales with particle size to the 6th power, and hence, light scattering hugely overemphasizes the larger aggregates. Thus, based on the stretched-exponential fit, the true aggregate-size probability distribution strongly peaks near zero (monomers) and falls off approximately exponentially (see Fig. S4†). We therefore conclude that analysis of the DLS intensity-correlation function with standard methods results in erroneous and nonsensical aggregate sizes. The signal-to-noise ratio of DLS is of course not sufficient for a reliable extrapolation to zero. However, the distributions shown in Fig. 1(d), show that the data are consistent with aggregates at least as small as ∼40–50 nm.

However, in the nanoelectrospray ionization-mass spectrometry data presented here, we find clusters ranging from dimers to oligomers (*n* ≤ 30) along with multiple-charged species, implying the presence of even larger aggregates. Such oligomers have a size of approximately 
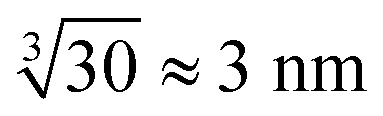
 leaving an unobservable gap from 3 to 40 nm. The mass spectrometry results are more extreme but consistent with previous observations of small (*n* ≤ 10) aggregates in undersaturated solutions of organic molecules.^44–46^ However, just as DLS is sensitive to the largest aggregates, mass spectrometry is prone to emphasize the smallest aggregates.

Phase-contrast microscopy places an even stronger emphasis on the largest aggregates. Thus, the submicrometre-scale aggregates observed by microscopy here must be at the very tail end of this wide distribution. These comparatively large aggregates are always observed near the edge of droplets likely deposited there through the coffee-ring effect.^47^

### The classical nucleation theory of aggregates can lead to nonclassical behaviour

The nucleation of aggregates takes place in a free-energy potential that is much shallower than that for crystal nucleation (× 10^−12^ in Fig. 5(b)*vs.*(d)). This implies that the kinetics of transformation toward larger aggregates will be slow and thermodynamic equilibrium will be reached slowly if at all. Indeed, our time-dependent DLS experiments (Fig. 1) show dynamics on a time scale of hours to days, while the size distributions (Fig. 1(d)) show lags and hysteresis.

At face value, the free-energy potential for aggregate nucleation shown in Fig. 5(d) predicts a single amorphous aggregate of *r* ∼300 nm in equilibrium. However, the barrierless nature (Fig. 5(e)) of the nucleation of aggregates—caused by reduced surface tension combined with the shallow free-energy potential—will instantly (homogeneously) produce oligomers of a vast range of sizes, as we have observed here. Hence, Ostwald ripening would be needed to produce a single large aggregate; however, the reduced driving force and hence the dominance of kinetic factors will conspire to make Ostwald ripening very slow. In fact, the absence of a barrier allows the existence of aggregates even in undersaturated solutions (Fig. 5(f)) as has been observed experimentally.^44,45^ This is in sharp contrast to classical nucleation of crystals directly from solution, where there is a clear distinction between monomers in solution *vs.* crystals (Fig. 5(c)). The existence of amorphous and partially ordered clusters on a range of length scales is consistent with computer molecular dynamics simulations on a wide range of systems.^48–51^

In the aggregates, the supersaturation with respect to crystal nucleation will be much larger than the initial supersaturation (*S*_0_). However, the interfacial tension associated with forming a crystal nucleus inside an amorphous aggregate remains large, resulting in a sizable barrier to crystal nucleation. In addition, effects such as vitrification or geometric frustration^49,52^ can give rise to additional thermodynamic and kinetic barriers to crystal nucleation. This is consistent with the experimental observation of slow crystal nucleation inside or on aggregates of small inorganic molecules^53^ and proteins^21^ and indeed with the results presented here.

The theoretical framework used here to describe our experiments differs from some previous nonclassical nucleation theories. The experiments show no evidence of liquid–liquid phase separation—suggested as a possible cause for nonclassicality^11,54^—even after storing samples for multiple weeks. They also show no evidence for assembly based pathways or oriented attachment,^5,6^ which is reinforced by the near instant nucleation upon laser irradiation. The experiments are consistent with a two-step process in which metastable amorphous clusters on a vast range of length scales nucleate slowly but homogeneously (barrierless). This is followed by nucleation of crystals in or on the amorphous clusters (Fig. 4) either spontaneously, induced by a laser, or through shearing.^43^ Thus, this is a two-step nucleation process in which each of the steps is itself classical hence leading to nonclassical behaviour as discussed previously.^38,55,56^

The key remaining questions concern the kinetics of the formation and relaxation of amorphous clusters, whether nucleation of crystals takes place in or on aggregates, and the supersaturation dependence of the nonclassical behaviour.^51,56^ The metastable but long-lived amorphous aggregates provide a window into the processes that inhibit the nucleation of the crystalline phase and may therefore be key to the development of amorphous drugs as well as providing insight into methods for polymorph selection. These results are an important step toward the full understanding of nonclassical nucleation pathways.

## Materials and methods

### Sample preparation

All amino acids (l-histidine, ≥99.5%, l-phenylalanine, ≥99.0%, l-proline, ≥99.0%, l-serine, ≥99.5%, l-glutamic acid, ≥99.0%, l-lysine, ≥98.0%, l-alanine, ≥99.0%, l-threonine, ≥98%, l-arginine, ≥98.0%) and peptides (Ala–Ala, ≥98.0% and Gly–Gly, ≥99.0%, Gly–Gly–Gly, ≥98.0%) were purchased from Sigma Aldrich and used as received. Supersaturated solutions were prepared by dissolving the amino acids and peptides either in H_2_O (Fisher Scientific, HPLC grade) or D_2_O (Sigma-Aldrich, 99.9%) in clean glass vials at 80 °C on a thermal shaker for 8 hours at a speed of 500 rpm and then gradually cooled to room temperature. pH/pD was measured using a PH8500-MS pH meter with a micro pH electrode/probe (Apera Instruments). All solutions were then filtered with Millex® PVDF filters with a pore size of 0.22 μm before DLS measurements and laser-induced nucleation investigation

### Dynamic light scattering (DLS)

Particle size analysis was carried out using a 647 nm laser and under precise temperature control using a particle size analyser (Anton Paar Litesizer 500) using a backscattering configuration (*θ* = 175°). Intensity autocorrelation traces were recorded, and initially, the standard cumulant method (Kalliope software) was used to analyse the autocorrelation decay curves and estimate the mean hydrodynamic diameter of the aggregates.

As the analysis with standard software did not fit the data well, we carried out nonlinear curve fitting (using Mathematica) with a stretched-exponential function, e^−(*t*/*τ*)^β^^. The stretched-exponential function can be written as a distribution of exponentials as
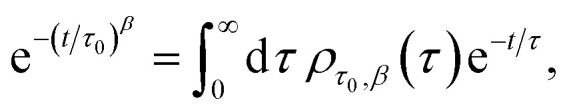
where *ρ*_*τ*,*β*_(*k*) is the relaxation-rate distribution function, which can be evaluated from the integral
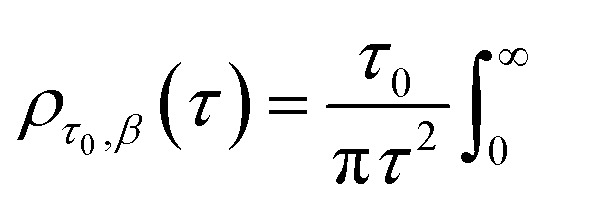

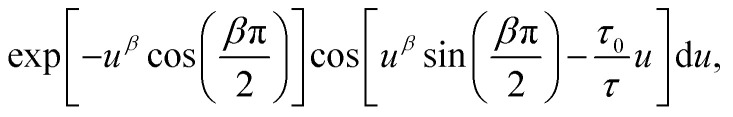
where *β* is the stretching parameter.^57^

The relaxation times, *τ*, may be related to a hydrodynamic radius using the Stokes–Einstein relation, that is,
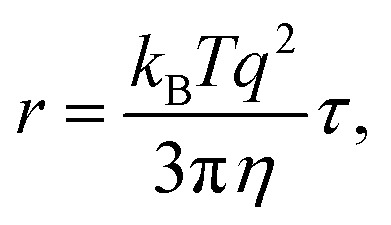
where *η* is the shear viscosity and 
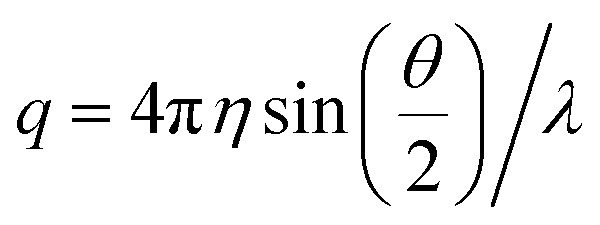
 is the scattering vector. The particle-size probability distribution functions *ρ*_*τ*_0_,*β*_(*r*), have not been corrected for size-dependent scattering.

### Mass spectrometry

Mass spectrometry was carried out on a Synapt G2Si instrument (Waters) with a nanoelectrospray ionization source (nESI). Mass calibration was conducted by infusing NaI cluster ions separately. Solutions were ionized from a thin-walled borosilicate glass capillary (i.d. 0.78 mm, o.d. 1.0 mm, Sutter Instrument) pulled in-house to an nESI tip with a Flaming/Brown micropipette puller (Sutter Instrument). A potential was applied to the solution using a thin platinum wire (diameter 0.125 mm, Goodfellow). The following instrument parameters were used for the Gly–Gly solution in H_2_O: capillary voltage 1.5 kV, source temperature 40 °C, trap collision energy 4.0 V, and trap gas 4 mL min^−1^. Sample cone and source offset were both set to 0 V for Gly–Gly–Gly and 40 V and 60 V, respectively, for Gly–Gly. Data were processed using Masslynx V4.2 and OriginPro 2021.

### Microscopy and Raman setup

A home-built setup for microscopy and Raman spectroscopy was employed on a double-deck inverted microscope (Olympus IX73). Phase contrast microscopy images and videos were taken using a 60×/0.7 N.A. objective (Olympus, UCPlanFL N Ph2) and a CMOS camera (Teledyne Dalsa, Genie Nano-1GigE). Two laser sources were simultaneously aligned into the microscope objective: a high-power pulsed 1040 nm laser (Spectra-Physics, Spirit One, 8 W) intended for optical tweezing and trapping and a single-frequency 532 nm laser with linear polarization (Laser Quantum, gem 532, 500 mW) for Raman excitation. Low frequency Raman spectroscopy was achieved using BragGrate™ bandpass and notch filters (OptiGrate), enabling detection of Raman scattering to frequencies as low as 10 cm^−1^. A spectrometer (Andor, Shamrock 500i with 600 groove per mm grating) and a CCD camera (Andor, iDUS 401) were utilized for detection. A confocal Raman collection employed an optical fibre with a 50 μm core size, and the spectral resolution of the system was approximately 2 cm^−1^.

### Data processing

All raw Raman scattering spectra were corrected (using MATLAB by MathWorks) with the Bose thermal-occupation factor, *I*(*ω*)/(1 + *n*(*ω*)), after background subtraction (dark counts of CCD detector), where *n*(*ω*) = (exp(−*ħω*/*k*_B_*T*) − 1)^−1^, *ω* is the angular frequency, and *I* is the Raman amplitude at *ω*, to obtain a reduced Raman spectrum.

As the Raman scattering signal strength from the small aggregates is weak, there is a relatively strong background from the glass microscope slides (as shown in Fig. S5†). All the spectra shown here have this background caused by glass subtracted.

## Data availability

The data that support the findings of this study are available in Enlighten: Research Data Repository (University of Glasgow) at http://dx.doi.org/10.5525/gla.researchdata.1671.

## Author contributions

KW, ZL, and AD conceived the experiments. CGR and AD conducted the mass spectrometry experiments, and RB analysed the results. ZL performed the DLS, Raman spectroscopy, laser-induced nucleation, and spontaneous nucleation experiments. KW developed the theoretical model. ZL and KW analysed the results and wrote the manuscript. All authors contributed to revising the paper.

## Conflicts of interest

The authors declare no conflicts of interests.

## Supplementary Material

SC-015-D4SC00452C-s001
